# A priori SNR estimation and noise estimation for speech enhancement

**DOI:** 10.1186/s13634-016-0398-z

**Published:** 2016-09-22

**Authors:** Rui Yao, ZeQing Zeng, Ping Zhu

**Affiliations:** College of Automation Engineering, Nanjing University of Aeronautics and Astronautics, Nanjing, China

**Keywords:** A priori SNR estimation, Noise estimation, Speech enhancement, MMSE

## Abstract

A priori signal-to-noise ratio (SNR) estimation and noise estimation are important for speech enhancement. In this paper, a novel modified decision-directed (DD) a priori SNR estimation approach based on single-frequency entropy, named DDBSE, is proposed. DDBSE replaces the fixed weighting factor in the DD approach with an adaptive one calculated according to change of single-frequency entropy. Simultaneously, a new noise power estimation approach based on unbiased minimum mean square error (MMSE) and voice activity detection (VAD), named UMVAD, is proposed. UMVAD adopts different strategies to estimate noise in order to reduce over-estimation and under-estimation of noise. UMVAD improves the classical statistical model-based VAD by utilizing an adaptive threshold to replace the original fixed one and modifies the unbiased MMSE-based noise estimation approach using an adaptive a priori speech presence probability calculated by entropy instead of the original fixed one. Experimental results show that DDBSE can provide greater noise suppression than DD and UMVAD can improve the accuracy of noise estimation. Compared to existing approaches, speech enhancement based on UMVAD and DDBSE can obtain a better segment SNR score and composite measure *c*_ovl_ score, especially in adverse environments such as non-stationary noise and low-SNR.

## Introduction

Single-channel speech enhancement has been used widely in various speech communication systems such as speech recognition, speech coding, and hearing aid devices. The main purpose of speech enhancement is to improve the quality and the intelligibility of speech. Spectral subtraction was the most widely used approach in early-stage speech enhancement applications, owing to the simplicity of implementation. However, it suffered from the unpleasant music noise. With the emergence of speech enhancement based on statistical models, a commonly used approach named as minimum mean square error (MMSE) spectral amplitude estimator was proposed by Ephraim and Malah in [1]. A priori signal-to-noise ratio (SNR) estimation and noise power estimation are key parameters in MMSE estimator, but accurate a priori SNR estimation and noise power estimation are not easy to obtain.

In order to estimate the a priori SNR, different solutions had been put forward [1–9]. Among them, the famous decision-directed (DD) approach proposed by Ephraim and Malah was based on the weighted sum of a priori SNR estimation in the previous frame and the a posteriori SNR in the current frame. In the DD approach, the weighting factor plays an important role in the performance of the algorithm, which shows the change of speech and is used to control the forgetfulness of the estimator. However, the weighting factor in [1] is set as a fixed value of 0.98, so the performance of speech enhancement is limited. Therefore, different approaches have been proposed to select the weighting factor. In [2], based on the assumption that additive noise is stationary and the noise energy does not change significantly from frame to frame, Soon and Koh proposed a low-distortion speech enhancement approach using an adaptive weighting factor. It works well for white noise but is less effective for non-stationary noise. Hasan et al. in [3] proposed a way to calculate the optimal weighting factor based on MMSE to account for the abrupt changes in the speech spectral amplitude. However, their approach cannot perform better than Ephraim and Malah’s due to some coupled reasons (for example, interaction of estimation errors). Cohen in [4] tried to calculate the weighting factor using future signal frames, and this kind of non-causal approach had a better performance than the causal one. Unfortunately, Cohen’s approach is always limited due to the additional delay. In [5], a technique based on the transient of the a posteriori SNR was proposed by Yun-Sik and Chang. The approach can reduce the delay and improve the segment SNR (segSNR) of a signal. Nevertheless, it cannot provide stable noise suppression and may introduce music noise because the dynamic range of weighting factor is too large. Except the DD approach, many data-driven and acoustic environment classification-based approaches had been proposed [6–8]. In [7], Choi and Chang used Gaussian mixture model (GMM) to identify the type of noise environment and then selected the optimal weighting factor according to the type of noise. This environment-sensitive scheme requires a substantial training process and is not robust under varying noise environments, even though it obtains a relatively good performance. These data-driven approaches can reduce speech distortion, particularly in speech onset; however, they also need a substantial training process to estimate a priori SNR. Recently, Lee and Chang proposed an approach based on multiple linear regression technique [9] which employed a real-time noise classification scheme based on GMM. However, their approach may not be reliable under complex acoustical environments because it depends on the accuracy of classification.

Among current approaches for a priori SNR estimation, the DD approach has a relatively acceptable performance with low computational cost. DD can reduce music noise effectively by providing smooth estimation of a priori SNR. However, as analyzed in [6, 9], DD often brings about roughly one-frame delay when it is used to estimate a priori SNR. What is more, the convergence rate of estimation is often slow because the weighting factor is close to 1, and the speech quality may seriously degrade when the delay is large. Under the ideal condition, the weighting factor *α* should be set as a small value in order to make sure that the a priori SNR estimation can rapidly change when the speech changes; otherwise, *α* should be assigned a value close to 1 providing lager noise suppression when speech is absent. And usually, the dynamic range of *α* should be restricted to avoid introducing music noise [10]. In addition, it is difficult to guarantee the estimation algorithm’s robustness in varying environments without the help of a special noise classifier, especially in a low-SNR environment. In brief, a robust feature to distinguish speech and noise is urgently demanded for speech enhancement.

Therefore, in this paper, a new DD approach based on single-frequency entropy named DDBSE is proposed, which combines DD and the approach in [1, 5, 11]. To overcome the drawbacks of the constant weighting factor adopted in DD, strong robustness of the energy entropy is utilized in DDBSE to identify speech and noise, and then, different *α*s are assigned to them. DDBSE can do well in adverse environments because the value of *α* only depends on the information of observations, without using any estimated parameter.

Noise power estimation is also a key factor in speech enhancement, which can usually be obtained through many approaches such as voice activity detection (VAD), minimum statistics (MS), and MMSE. In [11–16], VAD is the main research objective and is used to distinguish speech from non-speech. In these VAD-based noise estimation algorithms, the estimation of noise generally updates in the non-speech frame and remains unchanged in the speech frame. However, the accuracy of VAD cannot be guaranteed in low-SNR and non-stationary noise environments since a sudden rise in the noise power may be misinterpreted as a speech onset; moreover, the delay of noise estimation may be significant when the duration of speech is very long. Noise estimation based on MS estimates noise power level by observing the minimum discrete Fourier transform (DFT) coefficients of input signal in a small time window. Martin in [17] developed an unbiased noise estimator based on the optimally smoothed power spectral density estimate and the analysis of the statistics of spectral minima and proposed the MS-based approach for the first time. In [18], Cohen proposed a minima-controlled recursive averaging (MCRA) approach to calculate noise power, in which the speech presence probability of each frequency point is calculated by the relationship between statistical minimum and current input signal and is set as the weight of recursion. However, in most of MS-based approaches, too much residual noise induced by the under-estimation of noise will influence the speech quality, and the large delay caused by the time window, which may be as much as twice the length of the time window in the worst case, can decrease the accuracy of estimation significantly in varying noise background. Noise estimation based on MMSE supposes that the noisy speech is always in one of the two states, namely speech present (*H*_1_) and speech absent (*H*_0_), and adopts a recursion to estimate noise power rather than update estimation just when the state is *H*_0_ as the VAD-based approach does. In order to compensate for the bias caused by a priori SNR estimation in the traditional MMSE approach, Gerkmann and Hendriks in [19] proposed an unbiased MMSE-based noise estimator which used the a posteriori speech presence probability as the weight of recursion. MMSE-based approaches can update the noise estimation continuously and thus have no delay in theory. But they still have their own disadvantages, e.g., relying too much on precise a priori SNR estimation [20], easy to cause over-estimation of noise and thus damaging the speech, especially when speech continuously exists in a lot of frames.

Considering the advantage and disadvantage of noise estimation based on VAD [15] and MMSE [19], an unbiased noise estimation algorithm named UMVAD (noise estimation based on unbiased MMSE and VAD) is proposed in this paper. Similar to the MMSE-based approach, UMVAD takes the recursion that consists of the noise estimation in the previous frame and the observation in the current frame into consideration. Different from [19], UMVAD calculates the a priori speech presence probability (SPP) according to the change of entropy in every frequency point rather than uses a fixed value (0.5). In order to reduce the over-estimation and the under-estimation of noise, UMVAD also introduces the statistical model-based VAD [15]. By doing that, it is hoped that the algorithm can provide larger noise suppression when speech is absent and reduce the over-estimation of noise to protect speech when speech is present. In addition, UMVAD has modified the selection of threshold in the VAD algorithm, so as to solve the problem that the logarithmic mean of likelihood ratio is continuously greater than the threshold in some case. Finally, UMVAD will adopt different strategies in the silent segment and speech segment by making a decision between VAD-based and MMSE-based noise estimation. As experimental results show, the performance of UMVAD is better than those of VAD and MMSE.

The rest of the paper is organized as follows: Section 2 briefly reviews the principles of speech enhancement based on MMSE estimator, DD a priori SNR estimation algorithm, and the noise estimation algorithms based on MMSE and VAD. Section 3 introduces the proposed a priori SNR estimation algorithm DDBSE and the noise estimation algorithm UMVAD. Section 4 presents the experimental results and the discussion. Finally, a conclusion is given in Section 5.

## Review of basic principle

### Theory of speech enhancement based on MMSE

Based on the hypothesis that the noise is additive, the model of noisy speech in frequency domain can be expressed as

1 $$ Y\left(t,\kern0.5em k\right)=X\left(t,\kern0.5em k\right)+D\left(t,\kern0.5em k\right) $$

where *Y*(*t*, *k*), *X*(*t*, *k*), and *D*(*t*, *k*) represent noisy signal, pure speech signal, and noise signal, respectively; *t* is the frame index; and *k* is the frequency point. In general, the a priori SNR *ξ*_*t*,*k*_ and the a posteriori SNR *γ*_*t*,*k*_ can be defined as follows:

2 $$ {\xi}_{t,k}=\frac{\lambda_x\left(t,k\right)}{\lambda_d\left(t,k\right)} $$

3 $$ {\gamma}_{t,k}=\frac{{\left|Y\left(t,k\right)\right|}^2}{\lambda_d\left(t,k\right)} $$

In Eqs. (2) and (3), *λ*_*x*_ and *λ*_*d*_ represent the variance of pure speech signal and noise signal, respectively. The variance is equal to the power of a signal because the DFT coefficients of speech signal and noise signal are modeled as zero-mean complex Gaussian variables. Noise power is the square of the absolute of DFT coefficients. According to [1], the amplitude of the speech signal’s DFT coefficient ${\widehat{X}}_k$ can be estimated using Eq. (4), which can be derived based on the MMSE criterion.

4 $$ {\widehat{X}}_{t,k}=\frac{\sqrt{\pi }}{2}\frac{\sqrt{\nu_{t,k}}}{\gamma_{t,k}} \exp \left(-\frac{\nu_{t,k}}{2}\right)\left[\left(1+{\nu}_{t,k}\right){I}_0\left(\frac{\nu_{t,k}}{2}\right)+{\nu}_{t,k}{I}_1\left(\frac{\nu_{t,k}}{2}\right)\right]{Y}_{t,k} $$

5 $$ {\nu}_{t,k}=\frac{\xi_{t,k}}{1+{\xi}_{t,k}}{\gamma}_{t,k} $$

In Eq. (4), *I*_0_(·) and *I*_1_(·) represent the zero-order and the first-order modified Bessel function, respectively, and *v*_*t*,*k*_ is a function of a priori SNR *ξ*_*t*,*k*_ and a posterior SNR *γ*_*t*,*k*_ as is shown in Eq. (5). The speech signal in time domain can be restored by performing inverse Fourier transform (IFFT) on ${\widehat{X}}_{t,k}$, using the phase of noisy signal. As shown in Eqs. (4) and (5), the estimation of a priori SNR and a posteriori SNR are critical to speech enhancement.

### The DD approach for a priori SNR estimation

Usually, the DD approach proposed in [1] can be used to estimate a priori SNR *ξ*(*t*, *k*)

6 $$ \widehat{\xi}\left(t,k\right)=\alpha \frac{{\left|\widehat{X}\left(t-1,k\right)\right|}^2}{\widehat{\lambda}\left(t-1,k\right)}+\left(1-\alpha \right) \max \left(\widehat{\gamma}\left(t,k\right)-1,0\right) $$

where max(·) is the maximum function, *α* represents the weighting factor of recursion, and $\widehat{X}\kern0.5em \left(t-1,k\right)$ and $\widehat{\lambda}\left(t-1,k\right)$ represent the power spectrum estimation of clean speech and noise at the *t* − 1 frame, respectively. In Eq. (6), the first term represents the estimation of a priori SNR in the previous frame, and the second term is in relation to a posteriori SNR estimation. In [8], a minimum a priori SNR was proposed (*ξ*_min_ = − 15 dB), which can reduce the risk of introducing music noise. Weighting factor *α* shows the change of speech and determines the weights of both parts in Eq. (6). Without a doubt, weighting factor *α* is very important for the performance of a priori SNR estimation. By setting *α* as a fixed value closely to 1, the DD approach introduces nearly no music noise. However, it may lead to delay of estimation, since a fixed value cannot track the practical change of speech. In fact, in a non-speech frame, a large value of *α* close to 1 is beneficial to providing smooth estimation, while in a speech frame, especially when the change of speech is violent, *α* should be set as a small value in order to quickly track the change of speech.

### Noise estimation

#### Statistical model VAD-based noise estimation

VAD based on statistical model [15] detects whether speech is present or not in the current frame using the likelihood test criterion. According to [15], the DFT coefficients of speech and noise can be seen as asymptotically independent Gaussian random variables. And two hypotheses, *H*_0_ and *H*_1_, are adopted to represent speech absent and speech present, respectively. Then, the probability density functions conditioned on *H*_0_ and *H*_1_ are given by

7 $$ p\left(Y\left|{H}_0\right.\right)={\displaystyle \prod_{k=0}^N\frac{1}{\pi {\lambda}_N\left(t,k\right)} \exp \left\{-\frac{{\left|{X}_{t,k}\right|}^2}{\lambda_N\left(t,k\right)}\right\}} $$

8 $$ p\left(Y\left|{H}_1\right.\right)={\displaystyle \prod_{k=0}^N\frac{1}{\pi \left[{\lambda}_N\left(t,k\right)+{\lambda}_X\left(t,k\right)\right]} \exp \left\{-\frac{{\left|{X}_{t,k}\right|}^2}{\lambda_N\left(t,k\right)+{\lambda}_X\left(t,k\right)}\right\}} $$

where *λ*_*N*_(*t*, *k*) and *λ*_*X*_(*t*, *k*) are variance of noise and speech, respectively, |*X*_*t*,*k*_|^2^ is the DFT magnitude of speech, and *Y* is the observation (noisy signal) composed of all frequency points [15].

The principle of the statistical model-based VAD in [15] is as follows: firstly, the likelihood ratio of each frequency point Λ_*t*,*k*_ is calculated using Eq. (9); then, the logarithmic average value of all the frequency points is calculated and compared with threshold *δ* according to Eq. (10).

9 $$ {\varLambda}_{t,k}=\frac{p\left({Y}_{t,k}^2\Big|{H}_1\right)}{p\left({Y}_{t,k}^2\Big|{H}_0\right)}=\frac{1}{1+{\xi}_{t,k}} \exp \left\{\frac{\gamma_{t,k}{\xi}_{t,k}}{1+{\xi}_{t,k}}\right\} $$

10 $$ \frac{1}{N}{\displaystyle \sum_{k=1}^{N-1} \log {\varLambda}_{t,k}\underset{H_0}{\overset{H_1}{\begin{array}{l}>\\ {}<\end{array}}}}\delta $$

Parameter *δ* in Eq. (10) is always set as a fixed value, 0.15, so as to obtain a good performance [21]. And the result of VAD will be either *H*_1_ or *H*_0_. *H*_1_ means that speech is present (when the average value is greater than *δ*), while *H*_0_ represents that speech is absent (when the average value is smaller than *δ*).

Noise estimation based on VAD only updates estimation when speech is absent. It is reasonable because when speech is present, noise estimation will be equal to the estimation in the previous frame. Equation (11) [15] is used to update noise estimation according to the result of VAD above.

11 $$ {\widehat{\lambda}}_d\left(t,k\right)=\left\{\frac{\left(1-\beta \right)\cdot {Y}_k^2\left(t,k\right)+\beta \cdot {\widehat{\lambda}}_d\left(t-1,k\right)}{{\widehat{\lambda}}_d\left(t-1,k\right)}\kern1em \frac{\mathrm{when}\kern0.5em {H}_0}{\mathrm{when}\kern0.5em {H}_1}\right. $$

where ${\widehat{\lambda}}_d\left(t,k\right)$ represents the noise level (noise power spectra) and ${Y}_k^2\left(t,k\right)$ represents the power spectra of noisy speech (observation value). In Eq. (11), when speech is present (i.e., when *H*_1_), the estimation in the previous frame can be used; when speech is absent (i.e., when *H*_0_), in order to reduce the variance of estimation, noise power is updated according to the current observation ${Y}_k^2\left(t,k\right)$ and the previous estimation ${\widehat{\lambda}}_d\left(t-1,k\right)$. *β* is usually set closely to 1; here, it is equal to 0.98, which can obtain a satisfying performance.

As shown in Eq. (11), noise estimation based on VAD can cause heavy delay, especially when speech exists for a long time, because it is only updated when speech is absent.

#### Unbiased MMSE-based noise estimation

The VAD-based approach adopts hard speech presence probability, and it can only update noise estimation when speech is absent. The unbiased MMSE-based noise estimation of [19] modified the original MMSE-based estimator using the soft speech presence probability (SPP). This approach does not require bias compensation, and it can continuously update the noise estimation through the following recursive procedure. Firstly, the conditional expectation of noise power in frequency point *k* is computed using Eq. (12) [19].

12 $$ E\left(\left|{D}_{t,k}\right|{}^2\Big|{Y}_{t,k}^2\right)=\left(1-P\left({H}_1\left(t,k\right)\Big|{Y}_{t,k}^2\right)\right){Y_{t,k}}^2+P\left({H}_1\left(t,k\right)\Big|{Y}_{t,k}^2\right){\widehat{\lambda}}_d\left(t-1,k\right) $$

where $E\left(\left|{D}_{t,k}\right|{}^2\Big|{Y}_{t,k}^2\right)$ represents the conditional expectation of noise power in frequency point *k* under current observation ${Y}_{t,k}^2$, $P\left({H}_1\left(t,k\right)\Big|{Y}_{t,k}^2\right)$ represents the a posteriori SPP calculated by Eq. (13) [19], and ${\widehat{\lambda}}_d\left(t-1,k\right)$ is the noise estimation of the previous frame.

13 $$ P\left({H}_1\left(t,k\right)\Big|{Y}_{t,k}^2\right)\begin{array}{c}\hfill =\frac{p\left({Y}_{t,k}^2\Big|{H}_1\right)p\left({H}_1\right)}{p\left({H}_0\right)p\left({Y}_{t,k}^2\Big|{H}_0\right)+p\left({H}_1\right)p\left({Y}_{t,k}^2\Big|{H}_1\right)}\hfill \\ {}\hfill ={\left(1+\frac{p\left({H}_0\right)}{p\left({H}_1\right)}\left(1+{\xi}_{t,k}\right) \exp \left(-{\gamma}_{t,k}\frac{\xi_{t,k}}{1+{\xi}_{t,k}}\right)\right)}^{-1}\hfill \end{array} $$

Secondly, the noise estimation of the current frame ${\widehat{\lambda}}_d\left(t,k\right)$ can be calculated by Eq. (14) [19].

14 $$ {\widehat{\lambda}}_d\left(t,k\right)=\mu \cdot {\widehat{\lambda}}_d\left(t-1,k\right)+\left(1-\mu \right)\cdot E\left(\left|{D}_{t,k}\right|{}^2\Big|{Y}_{t,k}^2\right) $$

In [19], a priori SPP *P*(*H*_1_) and *P*(*H*_0_) in Eq. (13) are all set as a fixed value of 0.5, the level of a priori SNR *ξ*_*t*,*k*_ was set as 15 dB, and *μ* in Eq. (14) is set as 0.8.

Obviously, a posteriori SPP $P\left({H}_1\left(t,k\right)\Big|{Y}_{t,k}^2\right)$ only depends on the a posteriori SNR *γ*_*t*,*k*_, and it has an important impact on the result of Eq. (12). When noise is over-estimated, the a posteriori SNR *γ*_*t*,*k*_ can be small even though speech is present, and the a posteriori SPP $P\left({H}_1\left(t,k\right)\Big|{Y}_{t,k}^2\right)$ will become small too. The value of $E\left(\left|{D}_{t,k}\right|{}^2\Big|{Y}_{t,k}^2\right)$ in Eq. (12) may be seriously over-estimated when a posteriori SPP $P\left({H}_1\left(t,k\right)\Big|{Y}_{t,k}^2\right)$ is small and speech is continuously present because the result mainly depends on the value of noisy signal ${Y}_{t,k}^2$. In [19], a priori SPP and a priori SNR were set as a fixed value. This may cause noise over-estimation because it cannot provide correction to $P\left({H}_1\left(t,k\right)\Big|{Y}_{t,k}^2\right)$.

## Proposed approach

Entropy is commonly used to describe the amount of information provided by a signal. It relates the uncertainty of an event associated with a given probability distribution for a sequence of data. In general, the entropy of a series of data {*x*_1_, *x*_2_ … *x*_*N*_} can be calculated by Eq. (15).

15 $$ \mathrm{entropy}=-{\displaystyle \sum_{i=1}^N{p}_i\cdot \log \kern0.5em {p}_i} $$

where *p*_*i*_ is the probability density function of data *x*_i_. If the probability density function of each data *p*_*i*_ is approximately equal to the ratio of each data to the sum of data sequence, just as Eq. (16), the maximum entropy value will be obtained when all of the data *x*_i_ are equal, and a small entropy value will be obtained when the variance of the data sequence is large [22].

16 $$ {p}_i=\frac{x_i}{{\displaystyle \sum_{j-i}^N{X}_j}} $$

In most instances, especially to those voiced frames, the energy of speech sparser than noise, namely the absolute of DFT coefficients of noise (except periodic noise) (This paper takes no account of periodic noise.), is more balanced than that of the speech because those voiced frames are quasi-periodic in time domain and robust in noisy environments [21]. Therefore, according to the characteristics of entropy, the energy of the *R* continuous frames in each frequency point also follows: when speech appears in some of the *R* continuous frames, the entropy always becomes smaller than that in the case where all of the *R* continuous frames are noise frame. Therefore, it is reasonable to track the speech via the change of entropy. So in this paper, a modified DD approach based on the change of entropy (DDBSE) is proposed for a priori SNR estimation. Moreover, considering the importance of noise estimation for speech enhancement, a noise estimation approach based on unbiased MMSE and VAD (UMVAD) is presented.

### The DDBSE approach

DDBSE (DD based on single-frequency entropy) mainly modifies the weighting factor of DD in order to improve the accuracy of a priori SNR estimation. It is a fact that SNR can be high at a single frequency point when speech (especially voiced frame) is present, even though the overall SNR of a signal is low (such as 0 dB) [13]. To each frequency point, the entropy of the *R* continuous frames before the current frame will abruptly become small when speech suddenly appears in the current frame. Based on the analysis above, in a single frequency point, the change of entropy can be used to track speech. Therefore, DDBSE uses the change of entropy to calculate the weighting factor, so as to enable the weighting factor to adapt to the change of a signal.

**Fig. 1:**
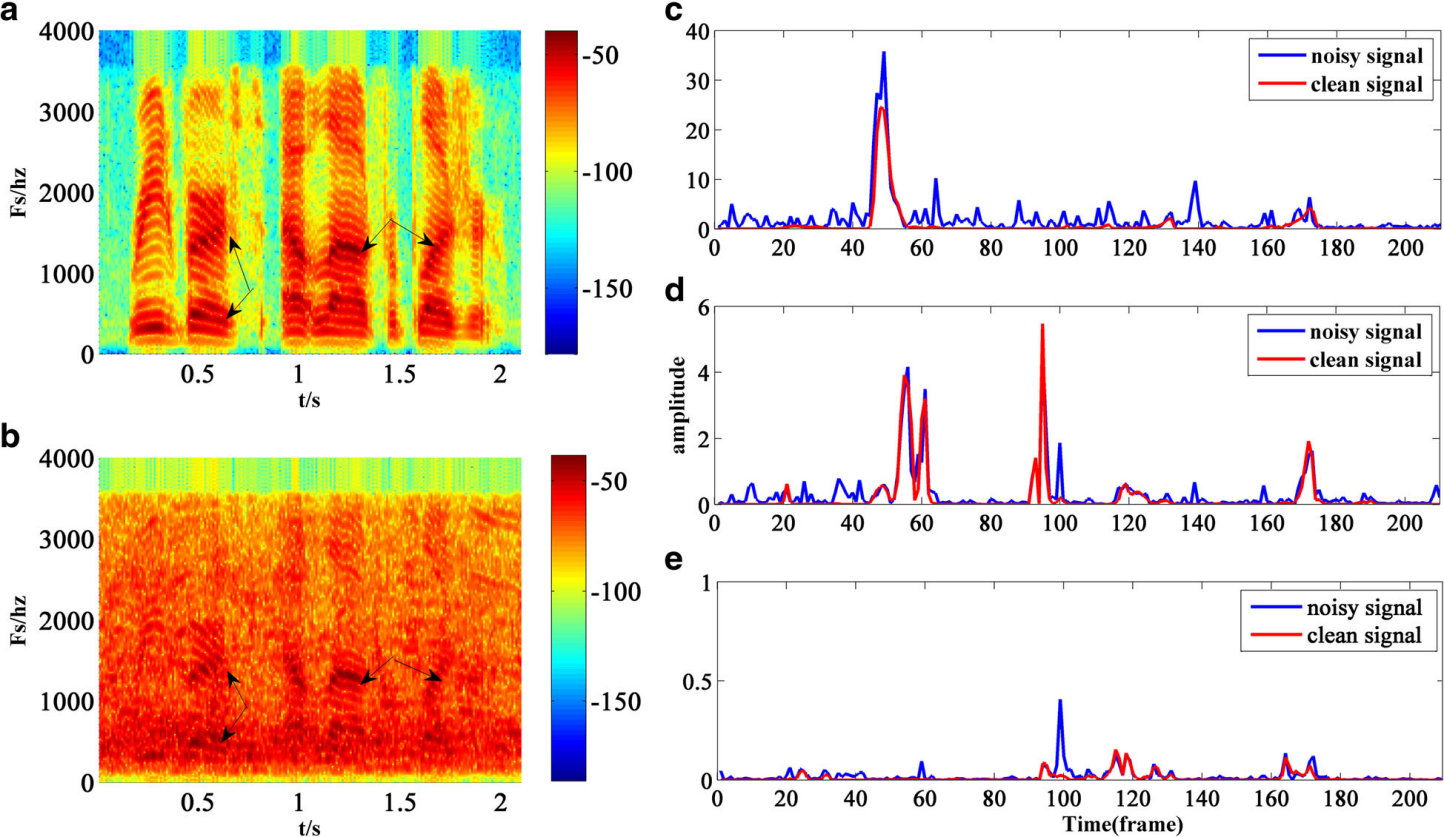
sp04.wav added by 0-dB babble noise. **a** Spectrogram of clean speech. **b** Spectrogram of noisy speech **c** when normalized frequency point *k* = 20, **d** when normalized frequency point *k* = 60, and **e** when normalized frequency point *k* = 120

For example, the sp04.wav selected from NOIZEUS corpus [23] are added by 0-dB babble noise and performed 320-point FFT, and the sampling frequency is 8000 Hz. The spectrogram of the clean speech and that of the noisy speech for the entire sentence are shown in Fig. 1a, b, respectively. In Fig. 1a, the harmonic structure of clean signal can be seen clearly, and the dark areas denoted by black arrows represent the harmonics with high energy. As can be seen from Fig. 1b, the harmonic structure and dark area can survive even in a low-SNR noisy environment. In order to better observe the change of energy distribution of clean signal and noisy signal, tree normalized frequency points *k* = 20, 60, and 120 (low, middle, and high frequency point) had been selected as an example. And the comparison of the DFT amplitudes between the noisy signal and the clean signal at normalized frequency points *k* = 20, 60, and 120 are shown in Fig. 1c, d, and e, respectively.

As can be seen from Fig. 1c–e, when *k* = 20, from frames 45 to 55, the speech is dominant in a signal; when *k* = 60, there are three areas dominated by speech, i.e., from frames 50 to 64, from frames 90 to 100 as well as from frames 165 to 175; when *k* = 120, the energy of speech is relatively small, so the energy distribution is even. Taking the area dominated by speech into account, we can conclude that the energy of speech mainly focuses on low and median frequency points, especially when the signal frame is voiced frame, so the SNRs in these areas are very high. Therefore, if these areas can be detected, the weighting factor can be set as a smaller value properly in order to quickly track the change of speech.

There are roughly four steps in DDBSE, i.e., global smooth, threshold process, entropy calculation, and weighting factor calculation.

Step 1: global smooth. The variance of noisy signal may be too large in case of low SNR. In order to reduce variance, the noisy signal should be smoothed before processing. This is done by Eq. (17).

17 $$ y\left(t,k\right)=\eta \cdot y\left(t-1,k\right)+\left(1-\eta \right)\cdot \mathrm{s}\mathrm{i}\mathrm{g}2\left(t,k\right) $$

where *y*(*t*,*k*) denotes the result after smoothing, sig2(*t*,*k*) denotes the original noisy signal, and when *η* = 0.85, the algorithm can obtain a good performance.

Step 2: threshold process. In DDBSE, the entropy of signal *y*(*t*,*k*) will be used to detect the speech frame. The entropy is calculated by considering the amplitudes of the *R* continuous frames in each frequency point. Just as Fig. 1 shows, although the energy of clean speech signal (denoted by a red line) is much greater than that in the frames nearby, there are still lots of interference caused by noise. In order to detect the speech frame more clearly, a local threshold processing-based approach is adopted, which processes the amplitude of the *R* continuous frames, tmp(*t*, *k*), as follows.

Firstly, the threshold thr is calculated by Eq. (18).

18 $$ \mathrm{t}\mathrm{h}\mathrm{r}=\rho \cdot \Big( \max \left(\mathrm{t}\mathrm{m}\mathrm{p}\left(t,k\right)- \min \left(\mathrm{t}\mathrm{m}\mathrm{p}\left(t,k\right)\right)\right) $$

In Eq. (18), tmp(*t*,*k*) represents the amplitude array of the *R* continuous frames before the current frame at frequency point *k*, and max (·) and min (·) represent the maximum function and the minimum function, respectively. And our experimental results show that a good performance can be obtained when parameter *ρ* = 0.6.

Then, each of the *R* values in tmp(*t*,*k*) is compared with thr to check whether it is less than thr or not. It will be set to 0 if the answer is yes; otherwise, it will remain unchanged.

Step 3: entropy calculation. After the process of step 2, the entropy of current tmp(*t*,*k*) can be calculated by Eq. (19). In Eq. (19), *R* = 5 and *c* = 0.12 [11]. *c* is fixed bias for logarithmic function.

19 $$ \mathrm{entropy}\left(t,k\right)=-{\displaystyle \sum_{n=1}^R\frac{\mathrm{tmp}\left(n,k\right)+c}{{\displaystyle \sum_{m=1}^R\left[\mathrm{t}\mathrm{m}\mathrm{p}\left(m,k\right)+c\right]}}}\cdot \log \left(\frac{\mathrm{tmp}\left(n,k\right)+c}{{\displaystyle \sum_{m=1}^R\left[\mathrm{t}\mathrm{m}\mathrm{p}\left(m,k\right)+c\right]}}\right) $$

**Fig. 2:**
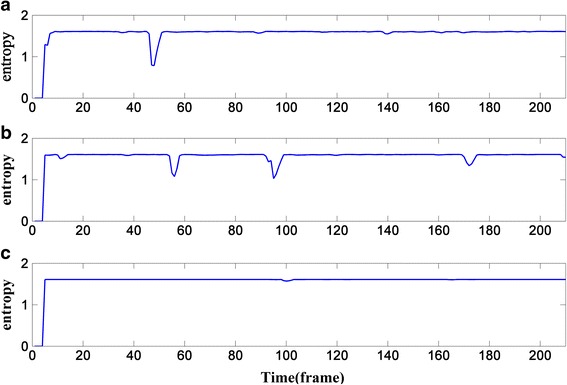
Entropy of the sp04.wav polluted by 0-dB babble noise in frequency point *k* = 20, 60, and 120. **a** When normalized frequency point *k* = 20. **b** When normalized frequency point *k* = 60. **c** When normalized frequency point *k* = 120

Figure 2 shows the change of entropy at the three frequency points corresponding to Fig. 1. The wave troughs in Fig. 2 indicate the wave peaks in Fig. 1, which represent the areas with strong energy. However, these regions with strong energy are always voiced frames. In addition, other areas in Fig. 2 are likely noise fames or low-energy speech frames, and their entropy is nearly at the same level.

Step 4: weighting factor calculation. The entropy of step 3 is used by DDBSE to calculate the weighting factor in DD. Here, the soft decision scheme rather than the hard decision scheme is adopted, as shown in Eq. (20).

20 $$ a\tilde{a}=\sigma +\mu \cdot \exp \left(\nu \cdot \left|\mathrm{entropy}\_\mathrm{now}-\mathrm{entropy}\_\mathrm{old}\right|\right) $$

In Eq. (20), DDBSE calculates an adaptive rough weighting factor *aã* by using exponential function similar to the approach in [5]; entropy_now and entropy_old represent the entropy of the current frame and the previous frame calculated by Eq. (19), respectively. A large number of experimental results show that the algorithm can obtain a satisfying result when *μ* = 0.0885, *v* = 5, and *σ* = 0.9.

**Fig. 3:**
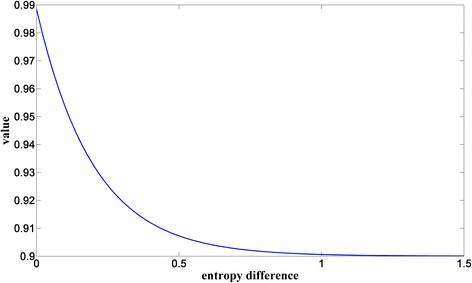
Dynamic range of the weighting factor

Figure 3 describes the dynamic range of the rough weighting factor calculated using Eq. (20). Obviously, the weighting factor can only change in the range of from 0.9 to 0.99. The risk of introducing music noise will decrease by doing that.

In order to obtain more stable a priori SNR estimation, the variance of weighting factor *aã* should be reduced further. Equation (21) is used to calculate the final weighting factor *aa*.

21 $$ aa(m)=0.7* aa\left(m-1\right)+0.3*a\tilde{a}(m) $$

where *aa* represents the final result and 0.7, 0.3 can obtain a good performance in experiments.

**Fig. 4:**
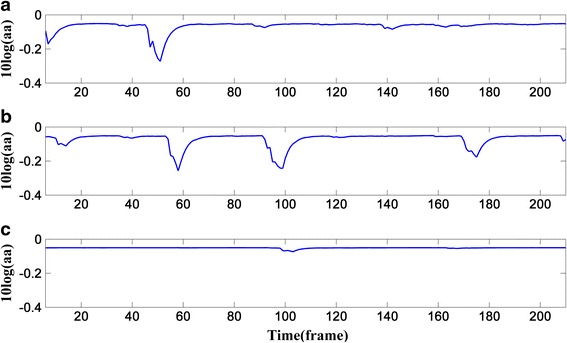
Change of the weighting factor’s logarithm: **a** the frequency point k=20; **b** the frequency point k=60; **c** the frequency point k=120

Figure 4 shows the change of *aa*’s logarithm at the three frequency points corresponding to Fig. 1. As can be seen from Figs. 2 and 4, the more violent the change of the entropy is in Fig. 2, the smaller *aa*’s logarithm will be in Fig. 4; and all the wave troughs in Fig. 4 are sharper than those in Fig. 2; all these can contribute to the convergence of Eq. (6), especially when *k* = 20 and 60.

**Fig. 5:**
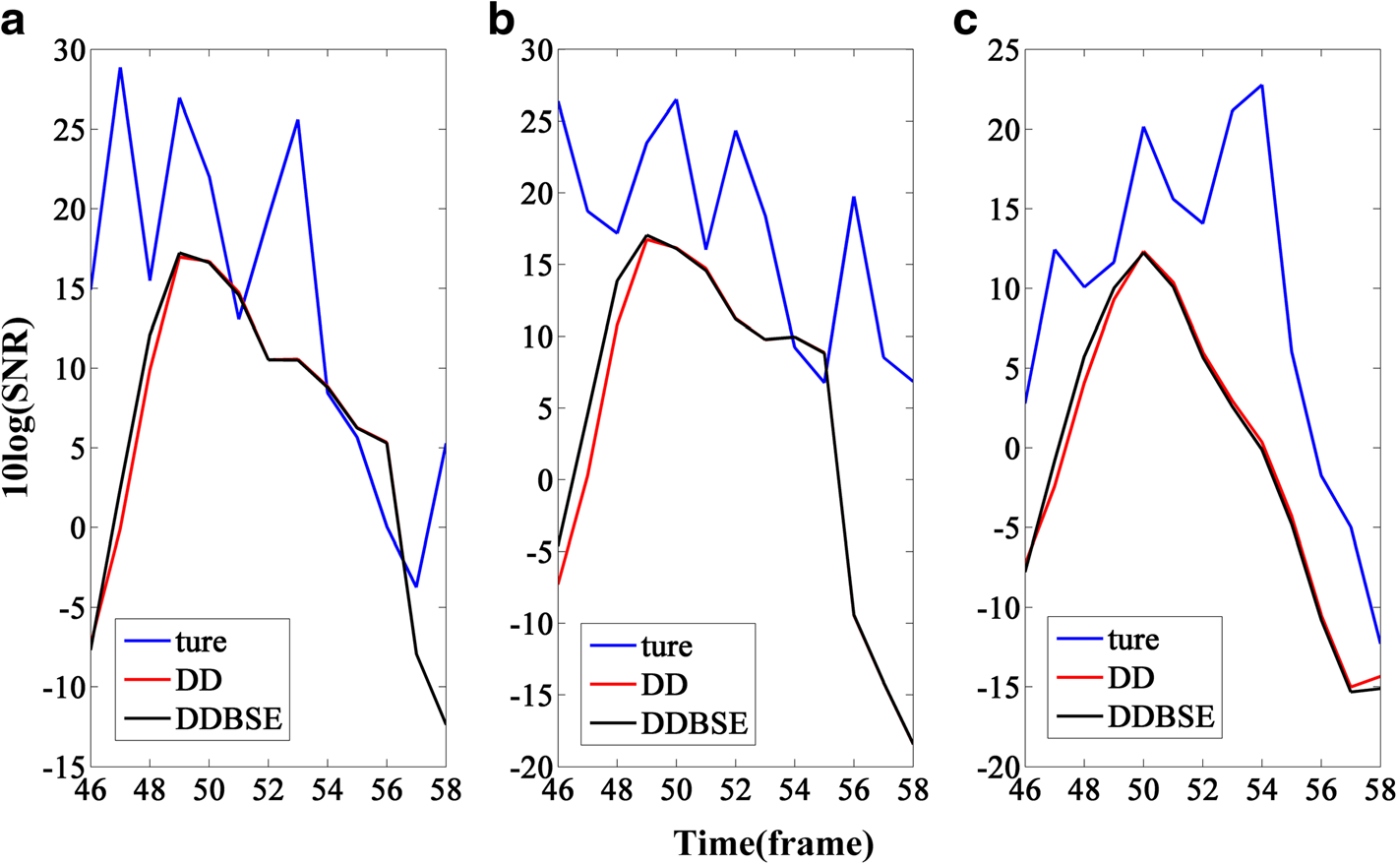
Comparison between DDBSE and DD. **a** Clean signal added by 0-dB car noise. **b** Clean signal added by 5-dB babble noise. **c** Clean signal added by 0-dB babble noise

Figure 5 shows the comparison of the a priori SNR estimation between DD and DDBSE. As can be seen from Fig. 5a, b, DDBSE can reduce the delay in the range of from frames 46 to 50, and that region represents the onset of speech. Therefore, DDBSE not only inherits the advantage of the DD approach but also can improve the performance of DD by reducing the delay. Even when the noise is low-SNR babble shown in Fig. 5c, DDBSE still can obtain a satisfying performance.

### UMVAD for noise estimation

Accurate noise estimation plays a decisive role in the intelligibility of speech [24]. However, most of the existing approaches for noise estimation suffer from the under-estimation and the over-estimation of noise, which make the intelligibility of speech hard to improve. In order to improve the accuracy of noise estimation, a new noise estimation approach named UMVAD is proposed in this paper, which is based on the statistical model-based VAD [15] and the unbiased MMSE [19, 20]. UMVAD adopts an adaptive threshold instead of the fixed threshold of VAD in [15] and utilizes the entropy to calculate an adaptive a priori SPP *P*(*H*_1_) to replace the fixed value of the unbiased MMSE in [19]. Moreover, UMVAD adopts different strategies to estimate noise according to whether speech is absent or not, so as to reduce the under-estimation and the over-estimation of noise.

#### VAD based on adaptive threshold

**Fig. 6:**
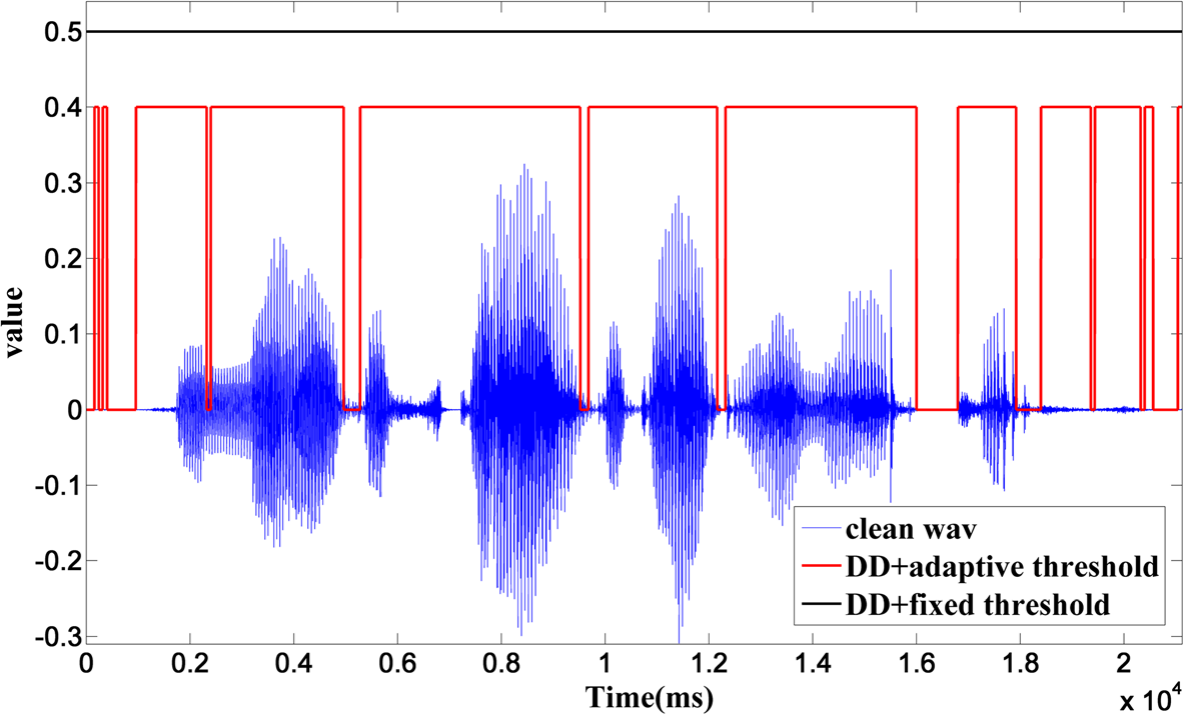
VAD result of sp02.wav added by 0-dB babble noise

Statistical model-based VAD in [15] is easy to operate and effective. However, in some cases, the average of likelihood ratio logarithm may be larger than the threshold in a long time because it uses a fixed threshold. Delay caused by that will seriously influences the performance of noise estimation. For example, in Fig. 6, the black line represents the VAD result of sp02.wav added by 0-dB babble noise using the approach in [15], and the result indicates that speech is absent in the whole time segment. If noise estimation only be updated in the silent segment, the delay may be as long as 2 s. Therefore, UMVAD proposes a new adaptive time-frequency threshold to replace the fixed one, as shown in Eq. (22).

22 $$ \delta (t)=0.15\cdot 0.8+0.2\cdot \frac{1}{10}{\displaystyle \sum_{i=t-1}^{t-10}\mathrm{v}\mathrm{a}\mathrm{d}(i)} $$

where vad represents the average likelihood ratio logarithm and *δ*(*t*) denotes the adaptive time-frequency threshold. In Eq. (22), the average vad of ten frames in front of the current frame is utilized to revise the original fixed value 0.15, and the decision result is relatively good when their weights are 0.8 and 0.2, respectively. As Fig. 6 shows, by using an adaptive threshold, the result of VAD (denoted by the red line) can find many silent segments that cannot be detected when a fixed threshold is used.

#### Calculation of a priori SPP

In [19], the unbiased MMSE-based noise estimation approach used fixed a priori SPP *P*(*H*_1_) to calculate the a posteriori SPP (*P*(*H*_1_|*y*)). As Eq. (13) shows, a posteriori SPP relies mostly on a posteriori SNR *γ*_*k*_. Once noise has been over-estimated, *γ*_*k*_ may become small, and (*P*(*H*_1_|*y*)) will become small too. What is more, noise will be seriously over-estimated because it mainly depends on the input signal ${Y}_{t,k}^2$ when speech is present. Therefore, the UMVAD approach proposes a new way to calculate *P*(*H*_1_) based on entropy, as shown in Eqs. (23) and (24). Even though that is not to match the definition of a priori SPP *P*(*H*_1_), this approach will make a priori SPP *P*(*H*_1_) more accurate.

23 $$ \overline{P}\left({H}_1(k)\right)= \exp \Big(\psi \cdot \max \left(1.61-\mathrm{entropy}(k),0\right) $$

24 $$ P\left({H}_i(k)\right)= \max \left( \min \left(\overline{P}\left({H}_1(k)\right),0.99\right),0.2\right) $$

**Fig. 7:**
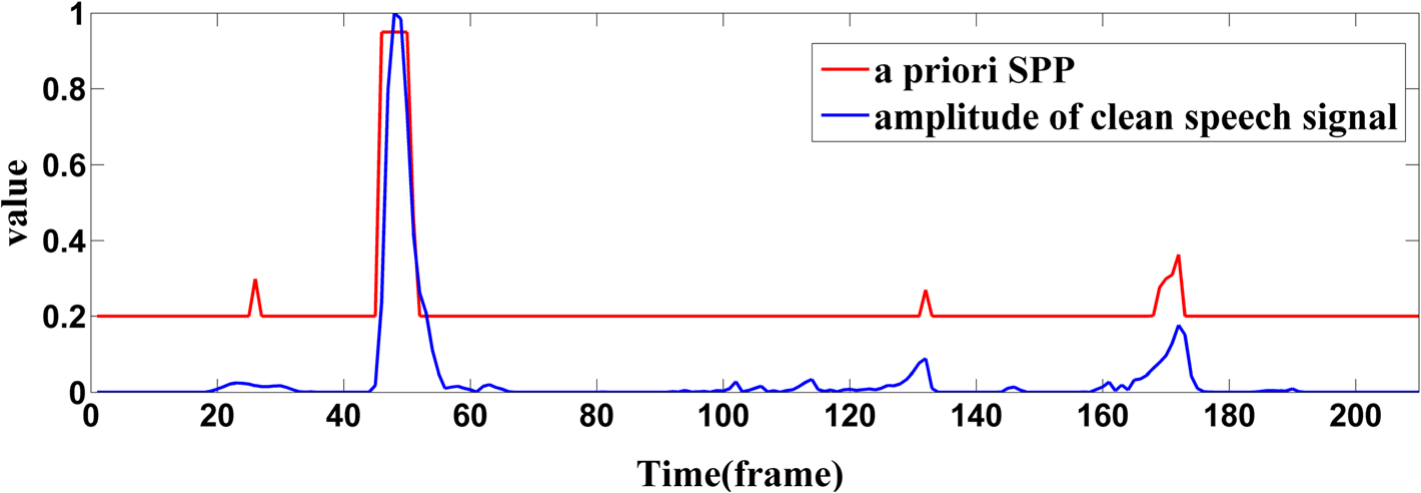
A priori SPP (*P*(*H*_1_)) of sp04.wav added by 5-dB white noise at normalized frequency point *k* = 20, and the amplitude of clean speech as comparison

In Eq. (23), entropy can be calculated by Eq. (19), and 1.61 is the maximum of entropy. *Ψ* is an adjustable parameter; here, it is equal to 2, with which algorithm can get a good performance. In order to prevent $\overline{P}\left({H}_1(k)\right)$ from being too small or too large, Eq. (24) can make *P*(*H*_1_) only change in the range of from 0.2 to 0.99. The reason for this is that the probability of speech presence may averagely be 0.2 in a long time [21] and the a posteriori SPP in Eq. (13) should be less than 1. Figure 7 shows the changes of *P*(*H*_1_) calculated by Eq. (24) and the normalized clean speech amplitude of sp04.wav added by 5-dB white noise at frequency point *k* = 20. As can be seen from Fig. 7, the value of *P*(*H*_1_) calculated by UMVAD may be large when speech is present, especially when these segments are voiced frames like frames 45 to 55; thus, a posteriori SPP (*P*(*H*_1_|*y*)) calculated by Eq. (13) will no longer become too large even if noise is over-estimated, when speech is present, while when speech is absent, UMVAD will provide small *P*(*H*_1_) and large (*P*(*H*_1_|*y*)) to protect the speech.

#### Strategy for noise estimation

In order to reduce the over-estimation and the under-estimation of noise, UMVAD uses different strategies to estimate and update noise. Firstly, the input noisy speech signals are classified into two categories: silent segments and speech segments according to the modified VAD based on the adaptive threshold described in Section 3.2.1. Then, aggressive strategy will be taken when speech is absent, while moderate strategy will be adopted when the current frame is a speech frame.

In silent segment, since the SNRs are different at different frequency points and the accuracy of VAD result is unsatisfying, in order to improve the accuracy of VAD, we classify all the frequency points into two categories further according to Eq. (25), and different strategies are taken on each of the two categories.

25 $$ \mathrm{i}\mathrm{n}\mathrm{d}=\left\{k\left|P\left({H}_1(k)\right)==0.2\&\&{\widehat{\xi}}_k\le 0.04\right)\right\} $$

where 0.2 and 0.04 are all empirical values that can obtain a good performance and ind represents the aggregate of frequency points *k* that satisfy the condition in Eq. (25). To these frequency points, noise estimation adopts the noise_mu2 in Eq. (25), where *E*(|*D*|^2^|*y*) represents the conditional expectation of noise power in Eq. (13). At the same time, to those frequency points which do not satisfy Eq. (25), noise estimation adopts noise_level calculated by Eq. (11).At last, noise_mu2 will be

26 $$ \mathrm{noise}\_\mathrm{m}\mathrm{u}2= \max \left(E\left(\left|D\right|{}^2\Big|y\right),\mathrm{noise}\_\mathrm{level}\right) $$

In speech segment, a conservative strategy will be used to estimate the noise in order to protect the speech. And the result noise_mu2 will be obtained from Eq. (27).

27 $$ \mathrm{noise}\_\mathrm{m}\mathrm{u}2= \min \left(E\left(\left|D\right|{}^2\Big|y\right),\mathrm{noise}\_\mathrm{level}\right) $$

## Experimental results and discussion

### Experimental environment

In this paper, 30 speech files from the NOIZEUS [23] corpus added by five kinds of noise are adopted to evaluate the performance of the proposed algorithms. These noises are babble, car, exhibition, station, and white, and all of them will be set as 0, 5, and 10 dB, respectively. All the algorithms are introduced into the MMSE-based amplitude spectrum estimator shown in Eq. (4), and the speech presence probabilities are utilized as the weights of the amplitude spectra [21]. In order to test the performance of these approaches, two objective measures, segmental SNR (segSNR) and composite measure *c*_ovl_ [25] will be used, where segSNR indicates the performance of denoising which is in relation to the quality of speech, while *c*_ovl_ (weighted sum of PESQ, LLR, WSS, and segSNR) has been regarded as a preferable measure about speech intelligibility. The purpose of speech enhancement is not only removing more noise but also reducing speech distortion. However, it is difficult to balance between denoising and speech distortion. The performance of speech enhancement based on DDBSE and UMVAD will be evaluated in the following sections. All the algorithms are implemented in MATLAB.

### A priori SNR estimation

In this section, the performance of the DDBSE approach will be tested. Both statistical model-based VAD and MMSE-based noise estimation have to use the a priori SNR estimation. Therefore, in order to test the performance of algorithm independently, the MCRA approach [26] and an easy recursive estimation shown in Eq. (28) rather than the statistical model-based VAD or the MMSE-based approaches are adopted for noise estimation. For all the algorithms, noise estimation is initialized by the average of the first six frames in each sentence, and segSNR is utilized as the measure of evaluation in this part.

28 $$ \mathrm{noise}\_\mathrm{m}\mathrm{u}2\left(t,k\right)=0.9\cdot \mathrm{noise}\_\mathrm{m}\mathrm{u}2\left(t-1,k\right)+0.1\cdot \mathrm{s}\mathrm{i}\mathrm{g}2\left(t,k\right) $$

In Eq. (25), noise_mu2 represents the final result of noise estimation and sig2 denotes input signal.

Tables 1 and 2 show the segSNR improvement comparisons of DDBSE and DD using MCRA and Eq. (28) for noise estimation, respectively.

**Table 1:** segSNR improvement comparisons of DDBSE and DD using MCRA

Noise	0 dB		5 dB		10 dB	
	DD	DDBSE	DD	DDBSE	DD	DDBSE
Babble	2.774	*2.885*	2.400	*2.519*	1.960	*2.021*
Car	4.300	*4.536*	3.831	*3.972*	3.050	*3.075*
Exhibition	3.558	*3.716*	3.102	*3.238*	2.434	*2.478*
Station	3.457	*3.637*	3.006	*3.132*	2.445	*2.468*
White	5.082	*5.367*	4.600	*4.771*	3.846	*3.850*
Average	3.834	*4.028*	3.388	*3.526*	2.747	*2.778*

The numbers in italics indicate the best performance. And all the results are the segSNR improvement compared to that of the untreated noisy speech

**Table 2:** segSNR improvement comparisons of DDBSE and DD using recursive estimation

Noise	0 dB		5 dB		10 dB	
	DD	DDBSE	DD	DDBSE	DD	DDBSE
Babble	2.533	*2.722*	0.661	*0.963*	−1.694	*−1.417*
Car	2.300	*3.364*	1.158	*1.509*	−1.15	*−0.930*
Exhibition	2.586	*2.857*	0.702	*1.006*	−1.687	*−1.411*
Station	2.707	*2.973*	0.825	*1.118*	−1.482	*−1.216*
White	3.187	*3.652*	1.369	*1.783*	−0.965	*−0.671*
Average	2.663	*3.114*	0.943	*1.276*	−1.396	*−1.129*

The numbers in italics indicate the best performance. And all the results are the segSNR improvement compared to that of the untreated noisy speech

As can be seen from Tables 1 and 2, DDBSE obtains a better performance than DD, whether MCRA or an easy recursive estimation is used. And the improvement is significant for stationary noises such as white and car. For example, when recursive estimation is adopted for noise estimation, DDBSE can obtain improvement up to 1 dB than DD does, and nearly 0.5-dB improvement for white noise. For non-stationary noises such as babble and exhibition, DDBSE also obtains improvement up to nearly 0.2 dB. Obviously, DDBSE can obtain a better segSNR than DD, and it means that DDBSE can provide a better ability of noise suppression owing to the more precise a priori SNR estimation. The reason for this is that the a priori SNR estimation provided by DD is smoother than that provided by DDBSE; thus, there is more residual noise due to the small aggressiveness of the DD approach.

A priori SNR estimation should make a balance between speech distortion and residual noise. In terms of the quality of speech, most listeners may think that overmuch residual noise is worse than a certain amount of speech distortion because noisy speech makes it easy to cause fatigue to the listener, especially in low-SNR and non-stationary noise environments. By introducing an adaptive weighting factor, the speech tracking ability of DDBSE has been enhanced, especially when speech starts or ends. Therefore, DDBSE can provide larger noise suppression than DD.

In addition, Tables 1 and 2 also show that MCRA can obtain a better performance than an easy recursive estimation. Moreover, it reveals that although a priori SNR directly impacts the performance of speech enhancement, accurate noise estimation is also important for the quality of speech.

### Noise estimation

Similarly, in order to test the performance of noise estimation algorithm independently, Eq. (29) recommended by [21] will be adopted.

29 $$ \mathrm{medSE}=\mathrm{median}\left({\frac{{\displaystyle \sum_k\left[{\widehat{\lambda}}_d^2\left(t,k\right)-{\lambda}_d^2\left(t,k\right)\right]}}{{\displaystyle \sum_k{\left({\lambda}_d^2\left(t,k\right)\right)}^2}}}^2\right) $$

where median denotes the median function. And the operational processes are as follows. Firstly, the difference between the noise estimation ${\widehat{\lambda}}_d^2\left(t,k\right)$ and the true noise power ${\lambda}_d^2\left(t,k\right)$ is normalized, and the sum of the square error at all frequency points is calculated further; then, the median of the sum values of all the frames in the current sentence, medSE, can be obtained; and the average medSE of all 30 sentences is viewed as the final result. Obviously, the smaller the average medSE is, the better the performance will be.

Table 3 shows the average medSE comparisons of the four noise estimation approaches: statistical model VAD, IMCRA [18], unbiased MMSE [19], and UMVAD.

**Table 3:** medSE comparisons of VAD, IMCRA, UMVAD, and [19]

Noise	0 dB				5 dB				10 dB			
	VAD	IMCRA	UMVAD	[19]	VAD	IMCRA	UMVAD	[19]	VAD	IMCRA	UMVAD	[19]
Babble	0.889	4.337	*0.599*	0.604	0.786	6.136	*0.611*	0.681	0.990	13.683	*0.738*	0.807
Car	0.538	3.416	*0.413*	0.483	0.541	1.699	*0.442*	0.540	0.577	53.179	*0.549*	0.616
Exhibition	1.039	5.750	*0.579*	0.614	0.675	1.878	*0.578*	0.665	0.853	16.509	*0.619*	0.717
Station	0.866	6.249	0.567	*0.539*	0.651	2.549	*0.563*	0.649	0.879	35.841	*0.648*	0.723
White	0.509	1.411	*0.436*	0.504	0.508	1.478	*0.445*	0.551	0.511	1.535	*0.463*	0.578
Average	0.761	4.263	*0.537*	0.558	0.660	2.684	*0.545*	0.628	0.756	20.853	*0.608*	0.715

The numbers in italics indicate the best performance

As Table 3 shows, VAD, [19], and UMVAD can obtain a good performance, while IMCRA performs worst owing to the under-estimation of noise. In all cases except station noise of 0 dB, UMVAD can obtain the minimum average medSE. It suggests that UMVAD can do better in estimating noise.

### Evaluation of overall performance

**Fig. 8:**
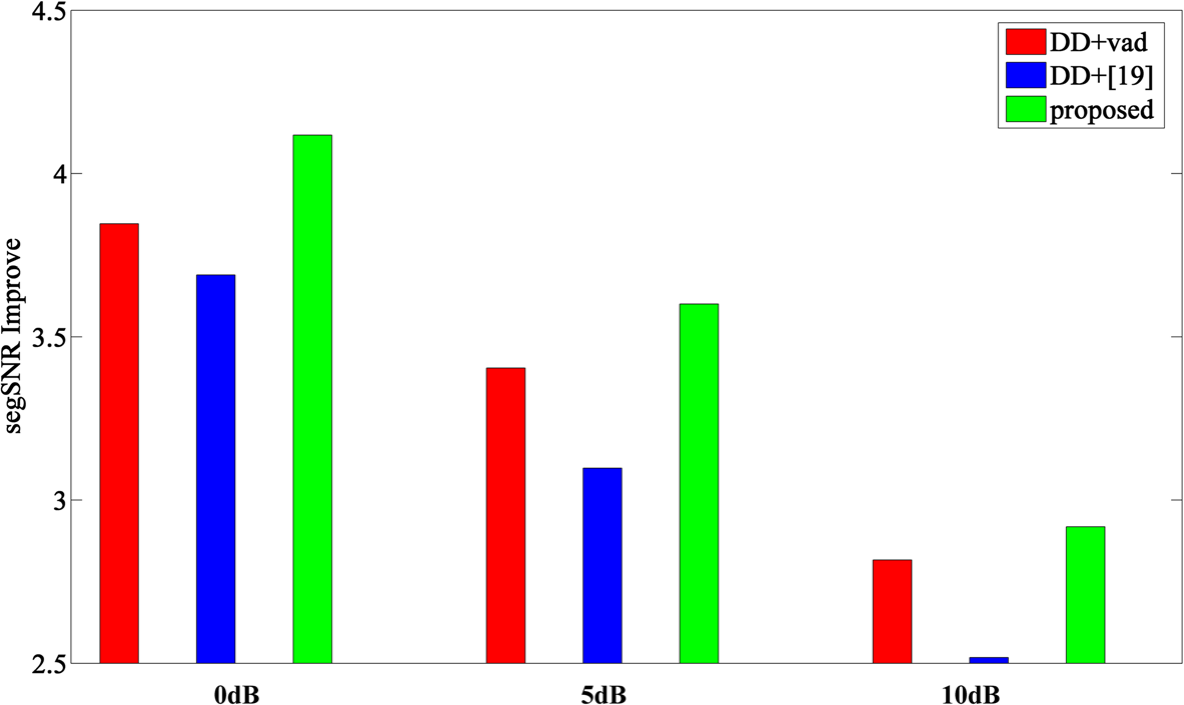
Comparisons of average segSNR

**Fig. 9:**
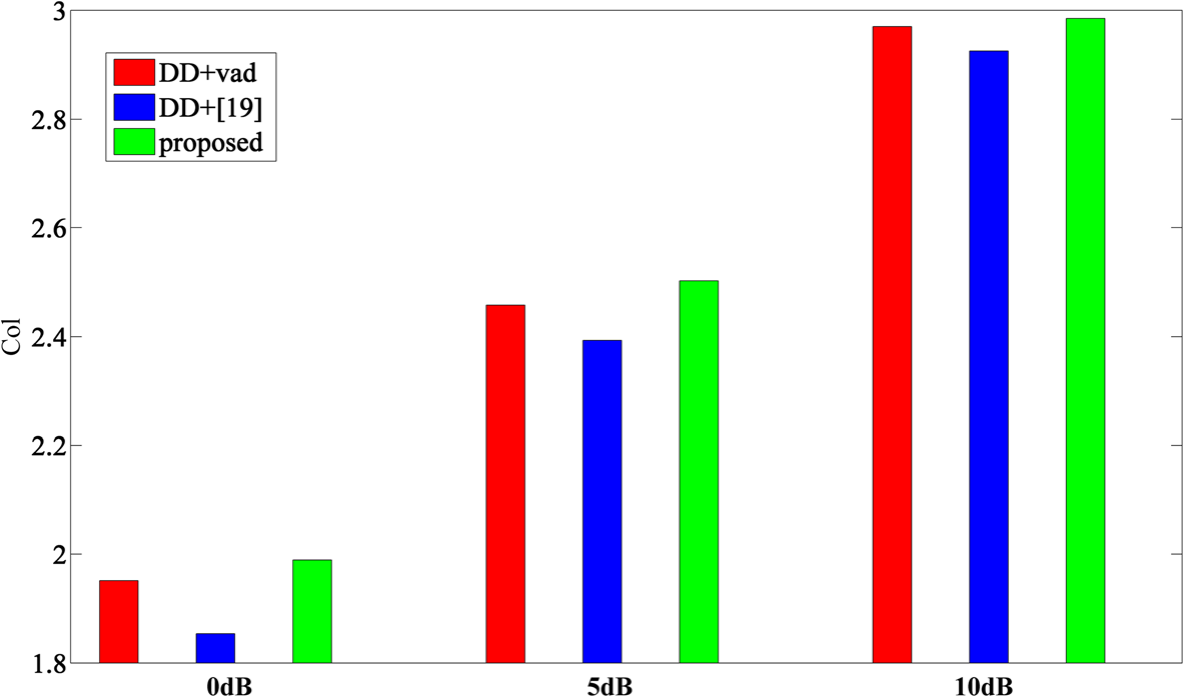
Comparisons of average *c*_ovl_ score

In Sections 4.2 and 4.3, DDBSE for a priori SNR estimation and UMVAD for noise estimation have been tested independently, and it has been proven that they can obtain a preferable performance. That is, DDBSE can provide more powerful capability of noise suppression, and UMVAD can estimate noise more accurately. In this section, the performance of statistic model-based speech enhancement based on DDBSE and UMVAD will be evaluated, and the speech enhancement based on DD and VAD (DD + VAD) as well as that based on DD and literature [19] (DD+[19]) will act as control groups. In order to evaluate the quality and the intelligibility of speech more profitably, both segSNR and *c*_ovl_ measures are used in this section, and the results are shown in Tables 4 and 5, respectively. In addition, the comparisons of the average segSNR and the average *c*_ovl_ of the three approaches are shown in Figs. 8 and 9, respectively.

**Table 4:** segSNR improvement comparisons of DD + VAD, DD+ [19], and proposed algorithms

Noise	0 dB			5 dB			10 dB		
	DD + VAD	DD+[19]	Proposed	DD + VAD	DD+[19]	Proposed	DD + VAD	DD+[19]	Proposed
Babble	2.552	2.797	*2.964*	2.204	2.190	*2.571*	1.774	1.594	*1.992*
Car	4.467	4.111	*4.649*	3.934	3.503	*4.085*	3.263	2.889	*3.219*
Exhibition	3.433	3.370	*3.764*	2.936	2.906	*3.324*	2.328	2.340	*2.703*
Station	3.532	3.472	*3.794*	3.057	2.802	*3.194*	2.506	2.150	*2.587*
White	5.245	4.702	*5.417*	*4.887*	4.088	4.833	*4.211*	3.617	4.091

The numbers in italics indicate the best performance. And all the results are the segSNR improvement compared to that of the untreated noisy speech

**Table 5:** *c*_ovl_ score comparisons of DD + VAD, DD+[19], and proposed algorithm

Noise	0 dB			5 dB			10 dB		
	DD + VAD	DD+[19]	Proposed	DD + VAD	DD+[19]	Proposed	DD + VAD	DD+[19]	Proposed
Babble	1.823	1.755	*1.883*	2.346	2.274	*2.398*	2.884	2.864	*2.927*
Car	2.216	2.092	*2.230*	2.674	2.571	*2.675*	*3.141*	3.068	3.114
Exhibition	1.799	1.726	*1.818*	2.327	2.331	*2.387*	2.859	2.876	*2.904*
Station	2.035	1.969	*2.058*	*2.636*	2.517	2.605	*3.056*	2.986	3.045
White	1.930	1.729	*1.958*	*2.505*	2.270	2.451	*2.989*	2.830	2.937

The numbers in italics indicate the best performance. And all the results are the *c*_ovl_ improvement compared to that of the untreated noisy speech

As Table 4 shows, for noises such as babble, car, exhibition, and station, the proposed approach provides higher segSNR than the other two approaches do; when noise is white of 5 and 10 dB, DD + VAD is a little better than the proposed approach. Overall, as Fig. 8 shows, the proposed approach denoted by a green bar can obtain a better performance. Particularly, when the noise is non-stationary, the performance of the proposed approach is much better than that of others. For example, the segSNR improvement about reluctant babble noise obtained by the proposed approach can be as much as 0.4 dB larger than that obtained by DD + VAD. It should be pointed out that the performance of most of the existing algorithms will degenerate significantly under non-stationary noise or low-SNR environments, even though they are fairly good under a stationary noise environment. Therefore, it is a challenge for speech enhancement under non-stationary noise and low-SNR environments. The experimental results show that the proposed approach is robust in these adverse environments thanks to the robust technique based on entropy. And it is also reasonable that while under a stationary noise environment (such as white, station, and car), the proposed approach does not obtain improvement as much as that in the adverse environment because the noise power is easy to estimate and the improvement space is relatively small.

As Table 5 shows, the *c*_ovl_ score of the proposed approach is obviously better than others when the SNR is low (0 dB), while the *c*_ovl_ score of DD + VAD is a little better when the SNR is high (5, 10 dB). Obviously, most of the algorithms can obtain a satisfying performance when the SNR is high or the noise is stationary. However, it is difficult to improve their performance in adverse environments. Moreover, the *c*_ovl_ score can be regarded as an objective measure highly correlated with the intelligibility of speech, and it is a very difficult work to improve the intelligibility of speech. Luckily, as it can be seen from Table 5 and Fig. 9, the *c*_ovl_ score of the proposed approach is slightly better than that of DD + VAD and DD+[19], especially under the adverse environment aforementioned.

On the whole, all the experimental results clearly show that both segSNR and *c*_ovl_ score of the proposed approach are relatively better than those of other approaches, particularly in low-SNR and non-stationary noise environments. And it indicates that speech enhancement based on DDBSE and UMVAD can provide a better trade-off between speech distortion and residual noise.

## Conclusions

A priori SNR estimation and noise power estimation are key factors in statistical model-based speech enhancement. Based on the classic DD approach for a priori SNR estimation, in this paper, we proposed the DDBSE approach according to the change of entropy at a single frequency point. DDBSE can provide a smaller weighting factor in the speech frame to adapt to the change of speech and adopt a larger weighting factor for noise suppression in the non-speech frame. Simultaneously, in this paper, we proposed UMVAD for noise estimation by taking statistical model-based VAD [15] and unbiased MMSE-based noise estimation [19] into account. UMVAD adopts an adaptive threshold instead of the fixed threshold of VAD, utilizes the entropy to calculate an adaptive a priori speech presence probability to replace the fixed value of the unbiased MMSE, and adopts different strategies to estimate noise according to whether speech is absent or not.

As experimental results show, DDBSE can provide larger noise suppression than DD, and UMVAD can improve the accuracy of noise estimation. DDBSE combined with UMVAD can obtain improvement in the quality and the intelligibility of speech, especially under non-stationary noise and low-SNR environments.

Of course, just as pointed out in [19], even though most of current approaches can obtain a satisfying performance in improving SNR of speech signal, the intelligibility of speech is still hard to improve. It is mainly because that the precise noise spectrum is very difficult to get. Therefore, in order to estimate noise spectrum, the most direct and effective way is to find some features that can do better in distinguishing speech frame from noise frame, and a better amplitude estimator should be taken into consideration, just as in [27, 28].
